# Liver Transplantation for Pediatric Liver Cancer

**DOI:** 10.3390/cancers12030720

**Published:** 2020-03-19

**Authors:** Rakesh Sindhi, Vinayak Rohan, Andrew Bukowinski, Sameh Tadros, Jean de Ville de Goyet, Louis Rapkin, Sarangarajan Ranganathan

**Affiliations:** 1 Hillman Center for Pediatric Transplantation, UPMC-Children’s Hospital of Pittsburgh, Pittsburgh, PA 15224, USA; andrew.bukowinski2@chp.edu (A.B.); sameh.tadros@chp.edu (S.T.); 2 Medical University of South Carolina, Charleston, SC 29403, USA; rohanv@musc.edu; 3 Mediterranean Institute for Transplantation and Advanced Specialized Therapies (ISMETT), 90127 Palermo, Italy; jdeville@ismett.edu; 4 Department of Hematology/Oncology, UPMC-Children’s Hospital of Pittsburgh, Pittsburgh, PA 15224, USA; louis.rapkin@chp.edu; 5 Department of Pathology, Children’s Hospital Medical Center of Cincinnati, Cincinnati, OH 45229, USA; sarangarajan.ranganathan@cchmc.org

**Keywords:** pediatric, liver cancer, liver transplantation, hepatoblastoma, hepatocellular carcinoma, liver sarcoma, neuroendocrine tumor, chemotherapy, PRE-TEXT, histopathology

## Abstract

Unresectable hepatocellular carcinoma (HCC) was first removed successfully with total hepatectomy and liver transplantation (LT) in a child over five decades ago. Since then, children with unresectable liver cancer have benefitted greatly from LT and a confluence of several equally important endeavors. Regional and trans-continental collaborations have accelerated the development and standardization of chemotherapy regimens, which provide disease control to enable LT, and also serve as a test of unresectability. In the process, tumor histology, imaging protocols, and tumor staging have also matured to better assess response and LT candidacy. Significant trends include a steady increase in the incidence of and use of LT for hepatoblastoma, and a significant improvement in survival after LT for HCC with each decade. Although LT is curative for most unresectable primary liver sarcomas, such as embryonal sarcoma, the malignant rhabdoid tumor appears relapse-prone despite chemotherapy and LT. Pediatric liver tumors remain rare, and diagnostic uncertainty in some settings can potentially delay treatment or lead to the selection of less effective chemotherapy. We review the current knowledge relevant to diagnosis, LT candidacy, and post-transplant outcomes for these tumors, emphasizing recent observations made from large registries or larger series.

## 1. Introduction

### 1.1. Historical Background 

Clinical liver transplantation (LT) was initially conceived as the treatment for unresectable liver cancer, and progressive cirrhotic liver disease. The first nine LT were attempted at three centers, worldwide, between 1963 and 1967 [1,2,3,4]. Of seven recipients in the US, six were adults with malignancy and included three with hepatocellular cancer (HCC), one with bile duct cancer, and two with metastatic colon cancer [1,2]. None survived beyond the fourth post-transplant week. The first recipient to experience extended survival was a 1.5-year-old girl with HCC who received LT on July 23, 1967 [5]. She survived 13 months before succumbing to metastases. The first 56 children to receive LT in the US between 1963 and 1974 included three with primary HCC, 40 with biliary atresia, and 13 with other cirrhotic liver diseases [6]. Among biliary atresia recipients, 36 were less than 6 years old, and four were older than 10 years. Three recipients with biliary atresia were found to have incidental HCC, and included two of four older recipients, and one of 36 younger recipients. The first US multi-center experience with LT for 12 hepatoblastoma (HB) cases from ten centers reported recurrence-free survival averaging 44 months (range 24–70) in 50% of recipients [7]. Recurrences in 3 of 12 patients (25%), all fatal, were associated with vascular invasion and a predominantly embryonal or anaplastic histology. The cluster operation, which included the stomach, pancreas, duodenum, and variable lengths of the jejunum in addition to the liver was proposed as curative resection for secondary liver involvement due to primary malignancies, such as ductal carcinoma and carcinoid [8,9]. Actuarial two-year survival was 35% and perioperative mortality was 25% in a series of 21 recipients. Durable benefit was reported with LT for metastatic endocrine tumors of the liver, after the primary lesion had been resected [10]. The lessons from these historical observations appear clearer in hindsight and have been confirmed over time.

LT would not have been successful without the enabling effect of chemotherapy, which provided much needed control of disease, established proof of unresectability, and confirmed chemosensitivity of primary and metastatic lesions, a reliable predictor of survival after LT. Cisplatin emerged as the main pre-LT chemotherapeutic agent showing greater efficacy in shrinking unresectable hepatoblastoma HB tumors, resolving pulmonary metastases, and increasing in the average interval of disease control in early series [11,12].

### 1.2. Overview, Challenges, and the Rationale for Informed Decision-Making

Pediatric liver cancer now accounts for a tenth of all pediatric LT in the United States. Roughly 95% of these LT are performed for HB, the most common pediatric liver cancer, and HCC, which is less common in children than adults. Therefore, treatment selection can be biased by expectations of high cure rates, which are the norm with chemotherapy and surgery for large HB tumors. Such outcomes are not seen with large HCC tumors. Alternatively, the expected high relapse rate for large tumors with vascular invasion is typical of HCC but not HB. Such generalizations can also influence treatment choices for the even rarer pediatric liver sarcomas like embryonal sarcoma, which have cure rates similar to HB, and rhabdoid liver tumor, which is relatively resistant to chemotherapy and prone to relapse after surgery. The rarity and highly skewed relative prevalence of pediatric liver tumors has unintended results. Diagnostic uncertainty, less effective chemotherapy, and delayed treatment are common. 

### 1.3. The Liver Transplant Option and Related Considerations

LT exchanges certain death due to liver cancer which remains unresectable despite chemotherapy, for the possibility of a cure, but a lifetime of immunosuppression. As with any other indication for LT, the decision-making requires establishing medical need, recognizing and managing associated co-morbidities to achieve surgical success that is expected in all patients, and assessing psychosocial support systems available to the child to ensure lifelong care. These elements provide an estimate of overall prognosis, expected patient survival that approaches or exceeds 85% at 10 years for most non-malignant indications, and reassurance that the liver graft, a societal resource, is being utilized responsibly. For the pediatric LT candidate with unresectable cancer, this decision-making process requires that every aspect of diagnosis, staging, and treatment be revisited knowledgeably.

## 2. Assessing LT Candidacy for Pediatric Liver Tumors

The pre-transplant evaluation of the cancer patient begins with a multidisciplinary “tumor board” comprising oncologists, pathologists, radiologists, and surgeons. Tumor histology is re-evaluated to determine whether the more common HB shares features with HCC or contains a high proportion of anaplastic cells. The variants may determine response to chemotherapy and outcomes. Imaging should establish anatomic boundaries, proximity to vessels, local progression on therapy, and extrahepatic disease, as described above. This imaging must be repeated within 30 days before performing the LT procedure. For the unresectable tumor close to the retro hepatic vena cava, where tumor margins are likely to be involved, a cava-inclusive whole liver graft, or a left lateral segmental liver graft which includes the donor cava, may be appropriate. The length of chemotherapy needed to establish persistent unresectability must not delay LT, because in the absence of a living donor, maintenance chemotherapy is still needed to control disease while waiting for a cadaveric donor. Prematurity is common in HB patients, and the associated bronchopulmonary dysplasia in some affected children may require prolonged ventilatory support. Associated cancer predisposition syndromes such as familial adenomatous polyposis in the child’s family will require frequent post-LT surveillance endoscopies by pediatric gastroenterologists, starting mid to late childhood.

## 3. Changing Tumor Incidence and Use of Liver Transplantation for Unresectable Liver Cancer

**Figure 1:**
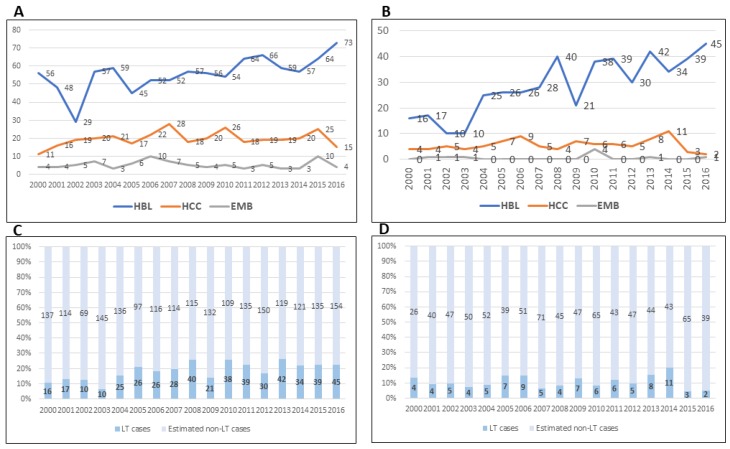
**(A**) HB, HCC and EMB (embryonal sarcoma) cases in the US. (**B**) Numbers of LT for HB, HCC and EMB in the US. (**C**) Estimate of HB cases treated with LT or other approaches. (**D**) Estimate of HCC cases treated with LT or other approaches. (SEER and SRTR 2000–2016).

The Surveillance, Epidemiology, and End-Results (SEER) program dataset reports cancer statistics from geographic regions representing 36.7% of the US population (SEER 21). Between 2000 and 2016, the numbers of subjects less than 20 years old with HB, HCC, and embryonal sarcoma were 983, 334, and 88, respectively. For the same time period, LT performed in the entire US for each of these tumors were 486, 95, and 9 respectively, in the Scientific Registry of Transplant Recipients (SRTR). The annual incidence of these cancers and LT for cancers (Figure 1) reveals notable trends.

In reviews of the SEER database, HB incidence increased significantly between 2004 and 2015 (annual percentage change, 2.2%; 95% confidence interval (CI) 0.5% to 3.8%, *p* < 0.05) [13]. In particular, this increase was observed among 2- to 4-year-old patients, males, and African-Americans. Among surgically treated patients, LT was performed in 17% of HB cases between 1998 and 2009 [14]. For the more recent period, 2004–2016, 21% or 93 of 443 surgically treated HB received LT, corroborating increased use of LT for HB [15]. This increase is further confirmed by extrapolating the SEER 21 incidence data based on 36.7% of the US population, to the entire population and calculating the proportions transplanted by using annual incidence of LT from the SRTR (Figure 1C and 1D). At least a fifth of all HB cases receive LT, corroborating yet another previous report [16].

Of 150 total HCC cases recorded during 2004–2015, 80 were treated surgically [14]. Twenty received LT, representing 25% of surgically treated HCC cases, or 13% of all HCC cases. The remaining cases received surgical resection. However, roughly 8–12% of estimated HCC cases in the US receive LT, a proportion that appears to have declined to 5% in 2015 and 2016. Viewed against the incidence of LT for HCC reported from an earlier time period from the SEER dataset by McAteer et al., it would appear that LT is applied variably or selectively in HCC cases [14]. Unlike the SEER database, which is current until 2016, the SRTR registry is more current, and records 12 LT in 2017 and 2 LT in the first half of 2018 for HCC. These additional data are also consistent with variable and possibly selective application of LT for HCC in recent years.

During 2000–2016, 9 LT were performed for embryonal sarcoma in the entire US. Extrapolating 88 cases of embryonal sarcoma reported in the same period from 36.7% of the US population (SEER 21) to 100% of the population yields 242 estimated cases, of which the 9 cases with LT in the SRTR database represent 3.75%. Other pediatric liver tumors are not described consistently in the two registries, precluding estimates of LT for rhabdoid tumors and metastatic liver tumors.

## 4. Presentation

In the SRTR database, 837 children have received primary LT for malignancy between 1987 and 2018, at a median age of 3 years, mean 5.1 years. These children include 499 males (60%), 693 Caucasians (83%), 72 African-Americans, and 72 children of other races. Demographics for each tumor are similar to those described in our previous review of 677 pediatric liver cancer patients who received LT in the US between 1987 and 2015 [17]. In that review, mean age at LT was lowest for HB (2.9 years) compared with HCC (12.8 years) or other categories of malignancy (range, 8.4–13.4 years). Male: female gender distribution was equal in HCC, skewed toward male distribution in HB, and female distribution in metastatic tumors and embryonal sarcoma. 

Liver cancer of childhood can occur with other birth defects, as described in the previous section, and elsewhere [18,19,20,21,22]. Reflecting previous observations, up to three quarters of HCC tumors can occur in liver that is affected by tyrosinemia, cirrhosis due to cholestatic and cirrhotic liver disease such as Biliary atresia or Alagille’s syndrome, familial cholestasis, viral hepatitis, and storage diseases such as Niemann-Pick Disease and ceroid lipofuscinosis [17].

**Figure 2:**
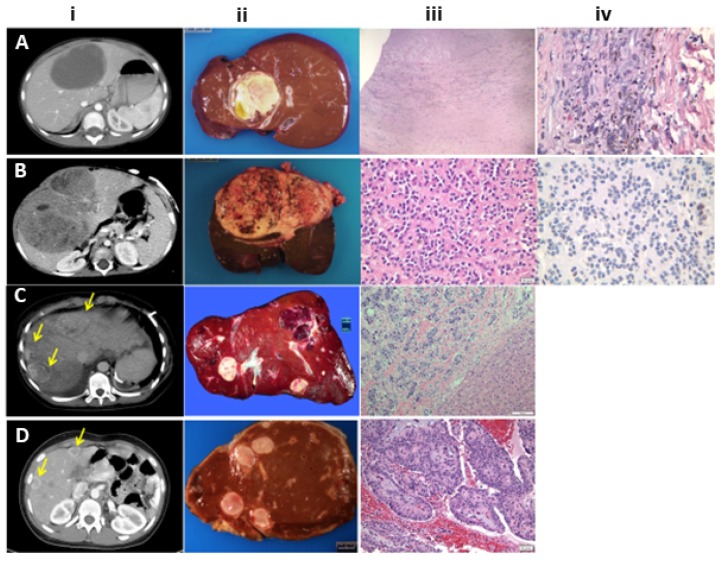
(**A**) First row: Undifferentiated embryonal sarcoma: i. Contrast-enhanced CT image with a hypodense mass in segment IV. ii. Liver explant with a large yellowish necrotic tumor. iii. H&E stain shows only a peripheral zone with atypical pleomorphic cells with abundant golden brown hemosiderin pigment in this treated tumor (×400). iv: H&E stain shows a necrotic tumor with areas of coagulative necrosis, cellular debris and fibrosis (chemotherapy effect) (×40). (**B**) Second row: Malignant rhabdoid tumor. i. Contrast-enhanced CT image show large hypodense solid mass with small areas of necrosis in both liver lobes. ii. Liver explant with large necrotic tumor. iii. H&E. Uniform tumor cells with abundant eosinophilic cytoplasm and eccentric nuclei with dense stromal sclerosis. (×200). iv. An INI1 immunostain shows the characteristic complete loss of nuclear staining (×200) (**C**) Third row: Metastatic neuroendocrine carcinoma. i. CT shows multiple hypodense rim-enhancing solid liver masses (arrows). ii. Multiple yellowish nodules of varying sizes characteristic of a metastatic tumor. iii. H&E stain shows the interface between normal liver and tumor arranged in nests with some pleomorphism of nuclei, dense stromal sclerosis and lymphatic invasion at the junction with the portal area in the center (H & E × 100). (**D**) Fourth row: Metastatic pseudopapillary tumor: i. Contrast-enhanced CT shows multiple rim-enhancing hepatic lesions (arrows). ii. Liver explant with multiple tumor nodules favoring a metastatic tumor in a patient with a known pancreatic primary. iii. H&E stain shows the classic pseudo-papillae of the primary pancreatic tumor with cells arranged around a myxoid matrix surrounding central vessels giving the tumor a papillary configuration. (H & E × 200).

Other presentations unique to children who are transplanted for liver cancer are the incidental finding of HCC at LT or during surveillance of underlying liver disease. Such tumors are likely to be early lesions with a favorable prognosis. Slow growing metastases from previously resected neuroendocrine tumors are detected in the course of routine post-surgical surveillance imaging. Embryonal sarcoma and rhabdoid liver tumor are sometimes confirmed during histological re-evaluation of liver removed at LT for an initial diagnosis of unresectable HB (Figure 2). Elevated alpha protein levels were present in 94% of patients with HB and nearly three quarters of patients with HCC in our published series of 75 children with LT for a variety of pediatric liver tumors. With a half-life of 6 days, alphafetoprotein (AFP) levels normalize within a few weeks after LT. Surveillance testing of AFP can detect recurrence.

## 5. Pathology

### 5.1. Hepatoblastoma

**Figure 3:**
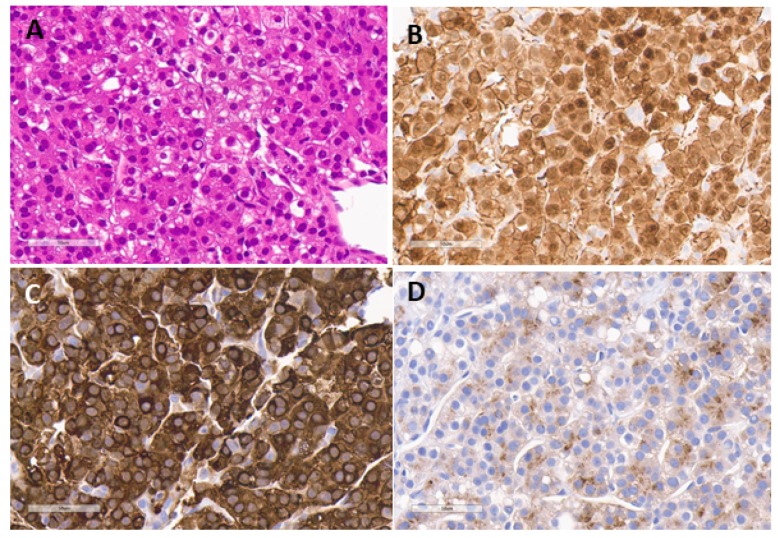
An example of HCN-NOS with overlapping features of HB and HCC in a 11 year old. (**A**) H&E showing a monotonous population of polygonal cells in a trabecular and pseudoacinar arrangement with variable sized nuclei and intranuclear vacuoles (H & E × 400). (**B**) beta-catenin stain shows a mainly cytoplasmic and weak to moderate nuclear stain throughout confirming a possible B-catenin mutation (B-cat × 400). (**C**) strong glutamine synthetase staining, thought to represent a surrogate marker of B-cat mutation (GS × 400). (**D**) Glypican 3 stain confirming the neoplastic nature of the lesion with fine granular staining of cytoplasm (GPC3 × 400).

The 2014 Consensus classification developed for HB is also useful for other liver tumors [23,24]. HB tumors are derived from pluripotent-stem-cell-derived hepatoblasts, which mostly differentiate into epithelial and mesenchymal elements, and sometimes, into neuroectodermal cells. A mesenchymal component is present in almost half of the HB tumors, which are designated mixed HB. The Epithelial element can be fetal or embryonal. The fetal component includes well differentiated fetal and “crowded” fetal (fetal with mitoses) histotypes and may be found either as a pure (100% well differentiated fetal type) HB, or part of a mixed HB, usually associated with high AFP. The pure well differentiated fetal HB (100%), with low mitotic count when completely resected, does not need adjuvant chemotherapy and is currently treated with observation alone. Embryonal HB is formed of more primitive cells and is considered low to intermediate risk, as is the crowded fetal subtype. Certain histologic patterns such as the macrotrabecular subtype (crowded fetal or embryonal cells arranged in thick trabeculae, at least 5 cells thick, mimics HCC) and is often associated with a higher stage disease. The Small cell undifferentiated (SCU) component is usually seen as small islands within embryonal areas of HB and does not adversely affect prognosis. A pure SCU tumor is more likely to be a malignant rhabdoid tumor and should be treated on a rhabdoid tumor protocol. Teratoid HB with neuroectodermal or primitive glandular components with yolk-sac-like appearance may behave aggressively if the latter components dominate. Immunohistochemical staining shows the hallmark beta-catenin nuclear staining in almost 90% of HB and is more pronounced in the more primitive forms [25]. Epithelial tumor cells are also positive for glutamine synthetase and glypican 3. Malignant Hepatocellular neoplasm not-otherwise-specified NOS (HCN-NOS) is a high-risk category which provisionally replaces the term ‘transitional liver cell tumor (Figure 3). Uniform cells without clear fetal or embryonal histology arranged in thin or thick trabeculae, with a high N:C ratio, may seem HCC-like in places. However, beta catenin staining and mutations are frequent along with many other acquired mutations, making these aggressive tumors [26,27].

### 5.2. Hepatocellular Carcinoma

Mostly arises in the setting of prior liver disease. The fibrolamellar variant occurs in a normal liver and is associated with a DNAJB1-PRKCA fusion transcript [28,29]. HCC tumors are sub-grouped into well-, moderately-, and poorly-differentiated categories similar to those in adults. Tumors may be discovered incidentally in the explant or detected during surveillance of underlying disease and trigger the decision to perform LT. HCC in children rarely show any beta-catenin nuclear staining. Nuclear beta-catenin should warrant a careful approach to rule out an HCN-NOS.

### 5.3. Malignant Rhabdoid Tumor (MRT)

This rare but high-grade malignancy of infancy may involve the liver or may be multifocal as part of the rhabdoid tumor predisposition syndrome. The tumor cells are round to polygonal cells with abundant dense eosinophilic cytoplasm with inclusions, large vesicular and eccentric nuclei, and numerous eccentric magenta colored nucleoli. These tumors do not produce AFP but are associated with mutations or deletions of the *INI1* or *SMARCA4* gene on chromosome 22 [30,31]. Thus, immunohistochemistry for INI1 is completely lost in the nuclei of the tumor cells but present in the nuclei of all normal cells.

### 5.4. Sarcoma

#### 5.4.1. Undifferentiated Embryonal Sarcoma of Liver

Undifferentiated embryonal sarcoma of liver (UESL) is another high-grade pleomorphic tumor of infancy with cells of varying shapes arranged in no particular pattern, high N:C ratio, and marked pleomorphism with numerous mitoses, including atypical ones [32]. A few tumors may show more hypocellular areas with myxoid change resembling mesenchymal hamartoma, a potential precursor lesion due to a common genetic defect in chromosome 19 (19q13.4). These tumors stain for vimentin, desmin, and CD10, and weakly for glypican 3. Absence of beta-catenin or myogenin differentiates these tumors from HB and rhabdomyosarcoma, while retained INI1 differentiates it from an MRT. Although these chemo-sensitive tumors can usually be cured with chemotherapy and surgery, LT is curative for selected cases when radical resection is not possible after chemotherapy [33].

#### 5.4.2. Rhabdomyosarcoma of Liver

Rhabdomyosarcoma (RMS) is a common malignant mesenchymal tumor in children that rarely can affect the bile ducts and the liver. Differential diagnosis with undifferentiated embryonal sarcoma may be difficult as they share some histologic features with spindle and polygonal cells in myxoid or mucinous stroma. Embryonal RMS usually affects the biliary tree as a polypoid intraluminal mass causing obstructive jaundice, that may infiltrate surrounding liver. Clinical and radiologic features and immunohistochemistry help to differentiate these from UESL (MSA, myogenin, and MyoD1 positive in rhabdomyosarcoma). This tumor is very sensitive to chemo- and radiotherapy and is often curable by standard resection with or without partial hepatectomy. Because the tumors arise from the bile duct and are often located at- or close to the biliary confluence, positive microscopic margin at resection is not rare. In these cases, radiotherapy may be proposed to obtain a cure and avoid local recurrence. In rare cases where radical resection was difficult to achieve, LT has been proposed to ensure a radical resection, and the authors argue that the radicality of the operation allows avoiding irradiation in a child and its long-term effects (scholastic effect, effect on growth and function of the regional organs, thoraco-vertebral deformities), which balances the need for long-term immunosuppression [34,35,36].

### 5.5. Vascular Tumors

Epithelioid hemangioendothelioma of the liver is an intermediate malignant tumor. Although the progression is very slow in adults who can benefit from LT, its behavior is more aggressive in children and is closer to that of angiosarcoma [37,38,39,40,41]. For that reason, it has been suggested in the past that LT should not be proposed in children; however, successful LT has been recently reported with a strategy of rapid LT in non-metastatic cases [42,43,44].

## 6. Radiologic Staging

Imaging plays a crucial role in staging and planning treatment of pediatric liver tumors such as HB and HCC, whether by LT or extreme resection [45]. Ultrasound is the initial screening modality for pediatric abdominal masses. Ultrasound provides a general assessment of tumor anatomy, its relationship to the portal vein, the hepatic veins, and the inferior vena cava and its limits, but may also identify subtle vascular invasion that is not visible on other modalities. Complete characterization of the mass requires detailed cross-sectional imaging such as computed tomography (CT) or magnetic resonance imaging (MRI). MRI is preferable for liver tumor evaluation because it has superior soft-tissue contrast, functional assessment provided by diffusion-weighted imaging, and hepatocyte-specific contrast agents, such as gadoxetate disodium (Gd-EOB-DTPA, Eovist/Primovist; Bayer, Leverkusen, Germany), which increase specificity and detection [46]. Today, MRI excels in the detection of vascular invasion by tumor. The capacity of multiple varied MR phases of contrast enhancement including dynamic post contrast three dimensional gradient echo (3D GRE) sequences and delayed hepatobiliary phase, with no additional radiation burden, is a significant reason why MRI is preferred by many radiologists [47]. Others have opted for CT-angiography, especially for large tumors and planning of extreme resections. Pre-operative post-CT processing with 3D-modelling and reconstructions also allows planning of surgical strategy and evaluation of resection planes [48,49]. Children usually require chest CT imaging because metastases from liver tumors affect the lungs, the most common site, in 10–20% of cases.

Risk stratification is undertaken with the Pretreatment Extent of Tumor (PRETEXT) system, originally proposed by the Société Internationale d’Oncologie Pédiatrique—Epithelial Liver Tumor Study Group (SIOPEL) for hepatoblastoma in 1992 [45,50]. The hepatic veins and portal veins divide the liver into its four sections: left lateral (Couinaud segments 2 and 3), left medial (segments 4a and 4b), right anterior (segments 5 and 8), and right posterior (segments 6 and 7). The PRETEXT group (I, II, III, or IV) is based on determining the number of contiguous tumor-free liver sections. PRETEXT I tumors have three adjoining sectors free of tumor, PRETEXT II tumors have two adjoining sectors free of tumor, PRETEXT III tumors have one sector free of tumor, and PRETEXT IV tumors have no sectors free of tumor. The PRETEXT groups are a powerful predictor of overall survival in children with HB and HCC. PRETEXT annotation factors such as V-vascular involvement (both hepatic venous and portal venous), E-extrahepatic disease, F-multifocal tumor and T-tumor rupture place C-caudate involvement, and N-lymph node metastases M-distant metastases each expand on risk stratification by denoting added risk, when present [51,52].

## 7. Chemotherapy

Given the rarity of pediatric liver tumors, collaborations have accelerated the development and standardization of chemotherapy regimens, which provide disease control to enable LT, and serve as a test of unresectability. For HB which is unresectable at diagnosis, both SIOPEL and Children’s Oncology Group (COG) recommend neoadjuvant chemotherapy with subsequent consideration of LT to ensure complete tumor removal. Until very recently, the chemotherapy approach utilized by each cooperative group differed both in chemotherapy agents utilized and scheduling [53,54,55,56]. The COG uses doxorubicin and 5-fluorouracil, and vincristine (C5VD) where the SIOPEL group has focused more on shorter frequency use of platinum agents with or without doxorubicin [53,54,55,56,57,58]. The SIOPEL group pioneered the use of POST-TEXT to reassess tumor burden after adjuvant chemotherapy. Following chemotherapy, conventional resection is recommended for patients with resolution of major vascular involvement and POSTTEXT I, II, or III group. Tumors which remain unresectable after chemotherapy are candidates for removal with LT. In some cases, aggressive nonanatomic resection with vascular reconstruction, and/or interventional embolization depending on the tumor extent and available expertise, may still be possible [59]. As discussed above patients with POST-TEXT IV and centrally located POST TEXT III have historically been considered candidates for LT; however, patients who have responded to chemotherapy and aggressive resection have been described in some series [59,60,61].

The Pediatric Hepatic International Tumor Trial (PHITT) is the first international cooperative liver tumor trial which will attempt to standardize the approach to chemotherapy for both HB and HCC by determining which regimen is most effective for treatment of these cancers [62,63]. This trial is important for patients in whom transplant is being considered as it will compare the event-free survival (EFS) with C5VD versus cisplatin monotherapy in patients with locally advanced non-metastatic disease. The study will also determine if EFS improves in patients with metastatic disease after treatment with an interval compressed regimen of cisplatin and doxorubicin, which was used in SIOPEL4, followed by a carboplatin/etoposide or vincristine/irinotecan treatment schedule.

In contrast with HB, HCC is also less sensitive to chemotherapy. In the SIOPEL 2 and 3 studies, 40% of cases responded to pre-operative chemotherapy with carboplatin combined with cisplatin and doxorubicin, without improvement in tumor resectability [64]. Overall 5-year survival was 22%, and included one survivor among seven with microvascular invasion, and two survivors among seven that received LT. Using either C5V or cisplatin/doxorubicin (PLADO) therapy in 46 HCC cases, the COG also demonstrated an overall 5-year event-free survival of 19% [65]. EFS was highest at 88% in stage I (resectable) tumors, 8% in stage III, and 0% in stage IV HCC. Thus, patient selection is essential to maximize survival. Patients with HCC and underlying disease will be monitored if a complete resection was obtained. The PHITT study will also determine the effectiveness of PLADO chemotherapy in patients with completely resected de novo HCC. In patients with metastatic disease, the study will evaluate the effectiveness combination therapies, for example, PLADO and sorafenib as well as PLADO with sorafenib alternating with Gemcitabine/Oxaliplatin and sorafenib.

Sarcoma and MRT: The treatment of embryonal sarcoma and MRT has developed from case reports and case series. Multimodal therapy consisting of local resection or LT, and neoadjuvant multiagent chemotherapy to facilitate resection, results in survival rates of 65–75%. Several regimens have included alkylators, platinums, podophyllotoxins, anthracyclines, and topoisomerase I. inhibitors, among others [33,66]. There is less agreement about the optimal treatment of MRT. Generally, very aggressive multimodal therapy is attempted, and includes surgery and radiation, despite the young age of these patients, as well as multiagent chemotherapy. Combination therapy with alkylators, anthracyclines, and platinums is common. Responses are limited and short lived in most patients, with 5-year survival rates approaching 30%, and most deaths occurring within a year of presentation [67]. Recently, Ribociclib, a CDK4/6 inhibitor in phase I trials, showed an increased rate of disease stabilization [68].

### Post-Transplant Chemotherapy

**Figure 4:**
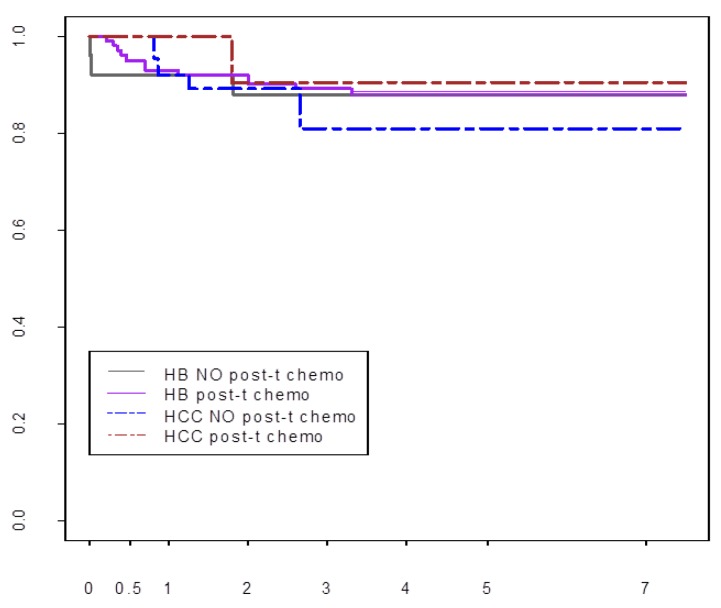
Pre-and post-transplant chemotherapy and survival after LT for HB and HCC. All 149 patients received three cycles of pre-transplant chemotherapy. Ninety six patients also received post-LT chemotherapy.

The international Pediatric Liver Unresectable Tumor Observatory (PLUTO) registry has collected outcome data of LT for liver cancer from 134 reporting centers [69]. These data do not show a survival benefit of adjuvant chemotherapy after LT in 110 HB patients, of whom 25 did not receive adjuvant chemotherapy, and in 39 HCC patients, of whom 28 did not receive adjuvant chemotherapy (Figure 4). However, these data must be interpreted with caution. The number of chemotherapy cycles is determined before LT and may be interrupted if a suitable allograft becomes available. The remaining cycles may then be given after LT.

## 8. Outcomes after Liver Transplantation

Reports based on the SEER registry have compared outcomes after surgical resection and LT, while those based on the SRTR database have described outcomes after LT for all liver cancers by era, and the effect of systemic changes on these outcomes [13,14,15,16,17,70]. These changes include assigning higher model-for-end-stage-liver-disease (MELD) or pediatric end-stage-liver-disease (PELD) scores for these tumors since 2010, the addition of doxorubicin and other agents to cisplatin-based COG protocols, AHEP-0731 (2009), and the use of PLADO with sorafenib for HCC (2007) [71].

### 8.1. Hepatoblastoma

Between 1998 and 2009, the SEER cancer registry recorded 318 surgically treated HB patients, of whom 17% received LT, and the remainder underwent resection. Survival was similar in the two groups. However, LT was performed for higher stage tumors and multiple hepatic satellite lesions [14].

**Figure 5:**
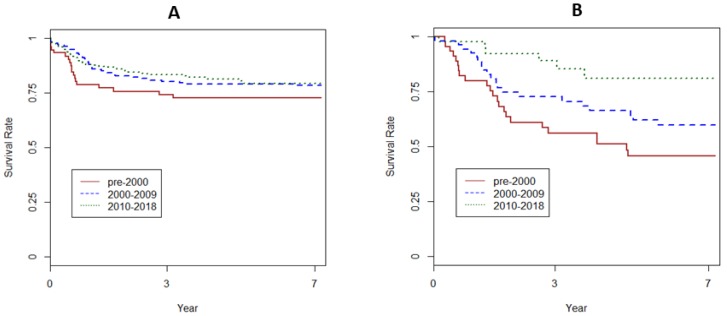
(**A**) Patient survival after LT for HB by era. (**B**) Patient survival after LT for HCC by era. (SRTR 1988–2018).

In the SRTR database, 5-year overall survival after LT for HB improved from 75.1% in the period before 2010, to 82.6% in the most recent decade. The waiting time also shortened, from 56.2 days to 33.2 days, *p* = 0.017, in the respective time periods [71]. Recurrent HB mostly occurs during the first two years after LT, has been associated with the presence of pre-LT pulmonary metastases and tumor necrosis < 50% after pre-transplant chemotherapy, and remains the most common cause of graft loss and death [16]. However, the anaplastic variant of HB was not associated with inferior post-transplant outcomes [16]. Patient survival by eras between 1988–2018 is shown in Figure 5A.

### 8.2. Hepatocellular Carcinoma

Of 150 total HCC cases recorded in the SEER registry during 1998–2009, 80 were treated surgically, and a higher percentage, or 25%, received LT [14]. LT was also associated with improved 5-year survival compared with resection in HCC patients, 85.3% versus 53.5%, and a significantly lower hazard of death (hazard ratio or HR, 0.05, *p* = 0.045). HCC treated with LT were likely to have vascular invasion and multiple satellite lesions. In this analysis of SEER data collected between 1998 and 2009 by., resected HCC tumors were more likely to be larger (11.3 cm versus 7.9 cm, *p* = 0.01), occur in older children (14.6 versus 10.7 years), associated with distant disease (21.7% versus 10.5%), compared with those who received LT [14].

In the SRTR database, 5-year overall survival after LT for HCC improved from 60% in the period before 2010, to 81% in the most recent decade [71]. The waiting time also shortened, from 56.2 days in the pre-2000 period to 33.2 days, *p* = 0.017, in the most recent period. Survival improved in each of the three decades, *p* = 0.008, Kaplan-Meier (KM) test (Figure 5B). Selection of lower stage HCC for LT may have also contributed significantly to these recent improvements [14]. The pre-1998 SEER database does not have such tumor characteristics, precluding additional analyses of a larger dataset to confirm our impression. In our review of SRTR data from 1988 to 2014, survival after LT for 22 children with HCC detected incidentally approached that of 3369 children with LT for biliary atresia (85% versus 89%, respectively, *p* = not significant or NS), the reference non-malignant indication for LT in children [17]. Although 10-year survival with incidental HCC was numerically higher compared with primary HCC (64% versus 40.5%, *p* = NS), the respective 95% confidence intervals of 71–100% versus 38–61% did not overlap. At our center, excellent survival outcomes were seen after LT for incidental HCC and HCC with preexisting liver disease, which led to early detection in our case series of 25 HCC [17]. No LT recipient with higher stage HCC or rescue LT for recurrent HCC which complicated previous major resection survived.

Whether pediatric HCC is biologically distinct from, and may have better outcomes than, adult HCC has not been resolved in a controlled setting. Some cytogenetic and molecular studies have found more similarities than differences between pediatric and adult HCC [72]. A comparison of 149 children and 15,714 adults with HCC who received LT for HCC did not show statistically significant differences in survival [17]. However, Milan criteria are not applicable to pediatric HCC. Of 13 tumors outside Milan criteria in our single-center series, which were 5 cm or more in greatest diameter, or presented with more than three lesions, five experienced durable recurrence-free survival after LT [17]. In the previously mentioned analysis of SEER data for 1998–2009, the average HCC tumor size in children who received LT was 7.5 cm, also outside Milan criteria [14]. Reflecting this uniqueness of pediatric HCC, tumor size outside Milan criteria was seen in five of 20 children in the SRTR dataset after 2010 [33]. Adding to the challenges of assessing LT candidacy is the fact that some pediatric HCC tumors which show extensive liver involvement are chemo-sensitive, remaining stable for months on chemotherapy. Yet other HCC tumors which were accepted for LT without reservations recurred late after LT, unlike the early recurrences seen after LT for HCC in adults. Therefore, decision-making must be individualized.

### 8.3. Primary Liver Sarcoma (PLS)

Outcomes after LT for PLS are less well understood, because of varying histologic subtypes, and a low incidence of no more than one in one million for any subtype [33,70]. The SRTR database lists these subtypes as embryonal sarcoma, rhabdomyosarcoma, and unclassified PLS (PLS-U). Malignant rhabdoid tumor (MRT), a distinct category of PLS, first appears in the SRTR database after 2000, because the characteristic loss of nuclear INI protein staining that distinguishes this tumor from other sarcomas was described after 2000 [30,31]. Between 1988 and 2018, 29 children with PLS received LT. No recurrences are recorded after LT for any of the 17 children with embryonal sarcoma. Four children each with RMS and PLS-U received LT. These subtypes were grouped together. In the pre-2000 period, recurrences occurred in three of four children with either RMS or PLS-U. No recurrences were seen in the four children who received LT after 2000, likely reflecting improved patient selection. Of three children with MRT, two died due to recurrence within a year after LT. No data are recorded after the day of LT for the third child. Therefore, LT must be undertaken with caution for unresectable MRT of the liver. Overall, however, LT for embryonal sarcoma and other subtypes of PLS is associated with excellent outcomes.

### 8.4. Metastatic Liver Disease

The outcome after LT for metastatic disease has been variable. Our analysis of SRTR data and single center experience both have shown excellent long-term outcomes [17]. As of 2018, the SRTR recorded 14 children with LT for metastatic disease, 10 with neuroendocrine tumors, of whom seven are alive. Of the remaining four children with metastases from neuroblastoma (*n* = 2), Wilm’s tumor (*n* = 1) and pancreatoblastoma (*n* = 1), three are alive after LT. Our single center series of 10 children with LT for metastatic liver tumors also reports 10-year survival of 67%. These outcomes suggest a favorable biology for tumors of childhood. A European transplant registry study of 213 adults aged 16–71, mean age 46 years, who received LT for metastases from neoroendocrine tumors, reported a 5-year overall survival of 52% and disease-free survival of 30% [10]. Predictors of poor survival were concurrent resectional surgery at LT and hepatomegaly, and age more than 46 years. Some of these negative predictors such as concurrent major surgery may be applicable to children. Extended follow-up is needed to confirm the durability of these outcomes.

## 9. Transplant-Related Care

The risk of vascular thrombosis may be higher in children with liver cancer, in part due to the thrombocytosis associated with HB, or to the use of cisplatin [16]. Low-dose heparin infusion of 2–5 units/kg/h initiated after arterial reperfusion, continued for the first five days after LT, and switched to prophylactic enoxaparin therapy for up to three months, may lower this risk. The mainstay of lifelong maintenance immunosuppression remains suppression of T-cell cytokine production with calcineurin inhibitors like tacrolimus. Steroids used early after LT are eliminated as tolerated in most pediatric recipients. Induction immunosuppression with T-cell-depleting agents, which is used in steroid-free regimens at our center, is contraindicated in the recipient with liver cancer, where anti-tumor T-cell-dependent surveillance mechanisms must be preserved to the extent possible. Fatal post-transplant recurrences have occurred in one recipient each after LT for HB and HCC in our early experience. Using lower dose immunosuppression in recipients with liver cancer immediately after LT, especially during post-transplant chemotherapy, is based on the belief that chemotherapy may impair anti-tumor T-cell-dependent immune surveillance. Lower rejection rates in recipients transplanted for liver cancer seen in some reports supports this view [73]. We have not observed such differences in rejection rates and thus do not alter our maintenance regimens [16]. However, chemotherapeutic agents, including cisplatin, facilitate tumor cell death by enhancing the anti-tumor response [73,74,75,76,77]. These observations support an immunosuppressive approach tailored to individual allograft function. Cancer surveillance includes serial monitoring of AFP for HB and HCC, and imaging for all tumors. The half-life of AFP is six days [78]. The frequency monitoring is highest during the first two years, when relapse is most likely, tapering to once annually until discontinuation after the fifth year.

## 10. Extreme Liver Resection and LT for Recurrent Disease

Extreme resections with close margins can be considered by expert surgical teams for those children ineligible for LT. In one series, six children, three with Pre-text IV involvement were treated with extended right hepatectomy in five and extended left hepatectomy in one, after six cycles of cisplatin chemotherapy, which also included doxorubicin in five, by a team consisting of experienced pediatric and liver transplant surgeons [79]. Two patients also required vascular reconstruction during the resection. Despite positive margins in two, and pulmonary metastases three months after surgery in one, all patients are alive at median 3.3 years (1.7–4.6). Approaches such as liver partition and portal vein ligation for staged hepatectomy (ALPPS), which have been used for extreme resections in adults, should be used with extreme caution. In one series, post-hepatectomy liver failure and sepsis developed in two of three adults with cholangiocarcinoma [80]. In a comparison of 11 adults treated with ALPPS and 54 treated with classical two-stage hepatectomy in another series, the future liver remnant underwent a greater rate of growth after ALPPS [81]. However, the functional liver remnant was smaller after ALPPS compared with classical two-stage hepatectomy. Rescue LT for relapse complicating a primary resection is associated with a higher relapse rate [82].

## 11. Genomic Landscape and Tumor Targeting

Clinical and histological features have limitations in assessing tumor response to chemotherapy and potential for relapse in individual patients. The tumor landscape may reveal novel druggable mutations, novel pathways for alternative chemotherapy, or changes in tumor clonality after primary chemotherapy that may help select more effective second line agents [83]. Unbiased tumor exome sequencing suggests that like many pediatric embryonal tumors, HB tumors harbor fewer mutations than tumors which affect adults [84]. Higher tumor mutation burden (TMB) renders tumors susceptible to immune therapy [85]. Among other findings, whole exome sequencing (WES) studies of HB implicate the well-known wingless integration-1 (Wnt)-beta-catenin signaling pathways, aberrant ubiquitin ligase signaling, and mutations that cause impaired function of tumor suppressors or critical transcription factors [86,87]. In a pan-landscape study of 24 tumors from nearly 914 children, the 16 HB tumors lacked novel druggable mutations [88]. In 31 chemo-resistant or metastatic HBL, four of whom required LT, a targeted panel of over 300 known cancer genes show a low tumor mutation burden (TMB) averaging 1.7 mutations/megabase of DNA sequence [87]. The study included a patient each with TMB > 10 and TMB > 20 mutations/megabase, a burden that is more often associated with tumors in adults. This panel also looks for specific mutations in *EGFR, ALK, BRAF, ERBB2,* and *BRCA1/2* genes which indicate response to specific agents in non-small-cell lung cancer, melanoma, breast, ovarian, and colon cancer. Anecdotally, this panel did not identify any such mutations in three children with recurrent HB after liver transplantation at our institution. One of these three children had an intermediate TMB of 9 mutations per megabase (muts/MB). One explanation may be that up to 65% of driver genes for pediatric cancers are distinct from those seen in adult cancers [89].

Tumor mutation analysis has other benefits. Circulating tumor DNA (ctDNA), measured as the proportion of circulating cell-free DNA that is derived from tumor, is an investigational tool to assess residual disease and response to treatment [90,91]. The components of this assay range from variants in 300–500 common cancer-associated genes (Guardant360 or Guardant Omni, Guardant Health), to custom panels that incorporate 16 unique clonal somatic variants individualized to each tumor (Signatera, Natera). ctDNA may be useful in assessing response to chemotherapy in HB which is associated with low alphafetoprotein levels. ctDNA may also distinguish tumor progression on chemotherapy from pseudo-progression [92]. Future benefits of the somatic mutation landscape may also include the identification of tumor-specific short and long peptide neoantigens which bind class I or class II major histocompatibility complex (MHC) molecules [93]. These neoantigens can be identified with computational analysis of non-synonymous single nucleotide variants. Once synthesized, the neoantigens can be used to generate patient-specific tumor vaccines and ex-vivo expansion of anti-tumor T-cells for cell therapy.

## 12. Conclusions

Total hepatectomy and liver transplantation is the only curative resection for primary liver cancer of childhood which remains unresectable despite neoadjuvant chemotherapy, and slow-growing metastases from neuroendocrine tumors which have been removed primarily. Patient selection has led to significant improvements in survival after LT for HCC, which is less sensitive to chemotherapy. These survival outcomes approach those for HB, the dominant liver malignancy which accounts for nearly a tenth of all LT in children. Among the subtypes of primary liver sarcoma, most of which have good outcomes after LT, the malignant rhabdoid tumor appears chemo-resistant and especially relapse-prone, and likely requires more effective neoadjuvant chemotherapy. Because lifelong anti-rejection medications and potential side effects are tradeoffs accepted by recipients with malignancy, the ideal future is a personalized approach in which novel approaches such as immune therapy or agents targeted to specific tumor mutations may hold the keys to a cure with minimal resection, or chemotherapy alone.

## Abbreviations

HB	hepatoblastoma
HCC	Hepatocellular carcinoma
SEER	Surveillance, Epidemiology and End-Results
SRTR	Scientific Registry for Transplant Recipients
AFP	alphafetoprotein
HCN-NOS	Hepatocellular neoplasm NOS
SCU	small cell undifferentiated hepatoblastoma
N:: C	nuclear to cytoplasmic ratio
PHITT	Pediatric Hepatic International Tumor Trial
SIOPEL	Société Internationale d’Oncologie Pédiatrique—Epithelial Liver Tumor Study Group
PRETEXT	Pretreatment Extent of Tumor
POSTTEXT	Post-treatment extent of Tumor
COG	Cooperative Oncology group
JCCG	Japan Children’s Cancer Group
MRT	malignant rhabdoid tumor
PLADO	Cisplatin and doxorubicin
C5VD	combination of cisplatin/5-flurouricil/vincristine/doxorubicin
EFS	event-free survival
PLUTO	Pediatric Liver Unresectable Tumours Observatory
TMB	tumor mutation burden
Mut	mutations
ctDNA	circulating tumor DN
MHC	major histocompatibility complex
